# Luxury in Surgery

**Published:** 1901-06-29

**Authors:** 


### Luxury in Surgery.

The remarks made by Lord Lister on the occasion of the opening of new operating rooms at St. Thomas's Hospital last week deserve the most careful attention of all who are engaged in the teaching of surgery. In these new theatres everything has been done that science can suggest to promote cleanliness and purity, so that aseptic surgery may be carried out in the most perfect manner. Lord Lister, however, while speaking with the highest appreciation of all he saw there, expressed a hope that his surgical friends would not misunderstand him when he said that he regarded such elaborate arrangements rather as luxuries than as essentials. "His own work," he said, "had been done in old-fashioned hospitals, and it was not for him to say whether his practice in those days had been satisfactory or not. But a day or two ago he had inspected the new operating theatre at the Western Infirmary at Glasgow, and there, while he saw much that reminded him of what was to be seen at St. Thomas's, the ventilation was on the old-fashioned system, there was no attempt to filter the atmosphere, no sterilisation by heat was practised, and the surgeons did not change their garments before entering the operating room. Yet as good results were obtained there as in any other place in the world." He had some jealousy, he said, lest students taught amid such luxuries as were afforded in the new theatres they had just seen might feel discouraged when performing operations in remote country districts, or in the homes of the poor, and so give up the attempt to get aseptic results, and he urged that those who were engaged in teaching surgery should make their pupils understand that good results might be obtained by simpler means. In truth, so far as theory goes, we believe they do. Every teacher of systematic surgery takes care that his students understand the object of each step taken, and the aims and limitations of each method employed in warding off septic infection. But the student is apt to be far more influenced by what he sees his teacher do in the operating theatre than by what he hears him say in the lecture room, and no one can doubt that a student who day after day becomes habituated to the use of all the elaborate appliances which are met with in some modern operating theatres, and becomes accustomed to see the operator surrounded by trained assistants who meet or even forestall his slightest want, comes by degrees to regard all these things as essentials, and to feel that surgery is beyond his reach until the happy time shall come when he shall obtain a hospital appointment. We think it just possible that, in speaking as he did, Lord Lister hardly bore in mind the extent to which the constant use of appliances which he described as luxuries conduces to the safety of the patients, to the elevation of the average standard of surgical work in a hospital, and to the ease with which that standard can be maintained. That Lister did good surgery, even when, as at Glasgow, he worked amid bad surroundings, no one doubts. But he did it by dint of a degree of personal care the necessity for exercising which stood for a long time in the way of the general acceptance of his methods, and although he may be right in speaking of many modern appliances as luxuries in that it is possible to do without them, they may also with propriety be regarded as necessaries in that they enable a standard of work to be attained as part of the ordinary daily routine of a hospital, and by the whole of the staff, which without these appliances would probably only be reached by the special enthusiasm of a few. This is to put our appreciation of modern aseptic methods and appliances at the lowest; but we are only expressing the opinion of many who have worked hard at the subject when we say that in the presence of that complete asepticism of patient and operator, of nurse and assistant, of instruments and dressings, of air and of theatre, which is the aim and object of these "luxuries," operations can be done which without them the surgeon would often hesitate to recommend.

Coming back, however, to the teaching of surgery, we can hardly doubt that the lessons learned from what one may call full-dress surgery are not those which are likely to be most useful to the ordinary student, who, in the great majority of cases, will hardly again enter an operating theatre after he once commences general practice. There is a surgery of the hospitals—a surgery of perfection for the few—and this is well taught. But there is also a surgery of the cottage—a surgery of "simpler means," and this, we fear, is in practice hardly taught at all. On every side we find the general practitioner "funking" surgery, and it is hardly to be doubted that he would have a good excuse were it to be held that for good surgery all the appliances of the modern operating room were essential. We do not like, with Lord Lister, to speak of these things as "luxuries," for we are sure that they have their uses. But we do think that Lord Lister is to be thanked for insisting that good surgery can be done with simpler means.

